# Muscarinic Receptors Types 1 and 2 in the Preoptic-Anterior Hypothalamic Areas Regulate Ovulation Unequally in the Rat Oestrous Cycle

**DOI:** 10.1155/2017/4357080

**Published:** 2017-03-14

**Authors:** Yadira L. López-Ramírez, Kayro López-Ramírez, Isabel Arrieta-Cruz, Angélica Flores, Luciano Mendoza-Garcés, Raúl A. Librado-Osorio, Roger Gutiérrez-Juárez, Roberto Domínguez, María Esther Cruz

**Affiliations:** ^1^Laboratory of Neuroendocrinology, Biology of Reproduction Research Unit, Facultad de Estudios Superiores Zaragoza, UNAM, Mexico City, Mexico; ^2^Department of Basic Research, National Institute of Geriatrics, Mexico City, Mexico; ^3^Division of Endocrinology, Department of Medicine, Albert Einstein College of Medicine, Bronx, New York City, NY, USA

## Abstract

Muscarinic receptors types 1 (m_1_AChR) and 2 (m_2_AChR) in the preoptic and anterior hypothalamus areas (POA-AHA) were counted, and the effects of blocking these receptors on spontaneous ovulation were analysed throughout the rat oestrous cycle. Rats in each phase of the oestrous cycle were assigned to the following experiments: (1) an immunohistochemical study of the number of cells expressing m_1_AChR or m_2_AChR in the POA-AHA and (2) analysis of the effects of the unilateral blockade of the m_1_AChR (pirenzepine, PZP) or m_2_AChR (methoctramine, MTC) on either side of the POA-AHA on the ovulation rate. The number of m_2_AChR-immunoreactive cells was significantly higher at 09:00 h on each day of the oestrous cycle in the POA-AHA region, while no changes in the expression profile of m_1_AChR protein were observed. The ovulation rate in rats treated with PZP on the oestrous day was lower than that in the vehicle group. Animals treated on dioestrous-1 with PZP or MTC had a higher ovulation rate than those in the vehicle group. In contrast, on dioestrous-2, the MTC treatment decreased the ovulation rate. These results suggest that m_1_AChR or m_2_AChR in the POA-AHA could participate in the regulation of spontaneous ovulation in rats.

## 1. Introduction

Acetylcholine (ACh) is the natural ligand of the muscarinic cholinergic membrane receptor (mAChR), which belongs to the superfamily of G protein-coupled receptors. There are five subtypes of mAChRs (m_1_ to m_5_). In particular, m_1_AChRs and m_2_AChRs have been detected in several brain areas; the highest proportion of m_1_AChR was found in the hippocampus, while the cerebellum and hypothalamus were the regions with the highest m_2_AChR expression [1].

Everett et al. and Markee and Hunter [2, 3] analysed the effects of blocking the muscarinic system with atropine sulfate, revealing that the cholinergic system plays a role in regulating the secretion of ovulating hormones in rats and rabbits. Moreover, intrahypothalamic atropine implants decreased ovarian weight and prevented ovarian compensatory hypertrophy in unilaterally ovariectomized rats [4, 5]. The injection of atropine in the 3rd ventricle blocked the surge of luteinizing hormone (LH) and follicle-stimulating hormone (FSH) by the pituitary gland in rats on the day of proestrous [6].

The muscarinic cholinergic system in the regulation of ovulation varies throughout the oestrous cycle and exhibits a circadian rhythm [7]. The subcutaneous injection of atropine sulfate on each day of the oestrous cycle blocked the preovulatory surge of LH on the proestrous day, without apparent changes in preovulatory FSH levels [8]. On the other hand, implants of atropine or pilocarpine crystals into the left or right side of the preoptic and anterior hypothalamus areas (POA-AHA) resulted in an asymmetric blockade of spontaneous ovulation, suggesting that the hypothalamic-muscarinic cholinergic system is involved in the asymmetric regulation of spontaneous ovulation and varies throughout the oestrous cycle [9, 10]. In recent studies [11], we showed that the unilateral microinjection of atropine in the left POA-AHA region performed on dioestrous-2 had the same effects as the implant of atropine in the same region.

The injection of synthetic LH-releasing hormone (LHRH-Gly-OH) or human chorionic gonadotropin (hCG) into nonovulating rats implanted with atropine in the right or left side of POA-AHA restored ovulation. Atropine implants placed in the right side, but not in the left side, of the POA-AHA on the day of oestrous or dioestrous-1 blocked the positive feedback of oestradiol benzoate (EB) on the release of gonadotropins necessary for ovulation. These results suggest that activation of mAChRs in the right side of POA-AHA plays a role in the oestrogen-dependent regulation of gonadotropin-releasing hormone (GnRH) preovulatory secretion [12]. Additionally, a unilateral implant of atropine in the POA-AHA modifies ovarian follicular growth in an asymmetric manner [13]. Taken together, these results suggest that depending on the day of the oestrous cycle and the side of the POA-AHA involved, the ACh binding to mAChR plays a stimulatory role in GnRH and LH preovulatory secretion and in the tonic secretion of FSH. However, according to Turi et al. [14], the classical cholinergic synapses rarely occur on GnRH neurons, suggesting a dominant nonsynaptic route in this cholinergic neuronal communication.

To assess the specific participation of m_1_AChR and m_2_AChR neurons located in the left or right side of the POA-AHA region on spontaneous ovulation during the oestrous cycle, we evaluated the following: (1) the variation of m_1_AChR or m_2_AChR protein on either side of the POA-AHA during each phase of the oestrous cycle and (2) the effects of the blockade of m_1_AChR or m_2_AChR during specific phases of the oestrous cycle on either side of the POA-AHA region. Based on these analyses, we intend to determine whether m_1_AChR or m_2_AChR in the POA-AHA is a trigger for the asymmetric effects of the muscarinic system on spontaneous ovulation.

## 2. Materials and Methods

### 2.1. Animals

The study was performed in 3-4-month-old virgin female rats (195–225 g) of the CIIZ-V strain from our own stock. The animals were kept under controlled light conditions (lights on from 05:00 to 19:00 h), with ad libitum access to regular chow (Harlan S.A., DF, México) and tap water. Oestrous cycles were monitored by cytological examination of daily vaginal smears. Only rats showing at least two consecutive 4-day cycles were used in the experiments. All microinjections were performed between 12:30 and 13:30 h.

### 2.2. Immunohistochemistry

**Figure 1:**
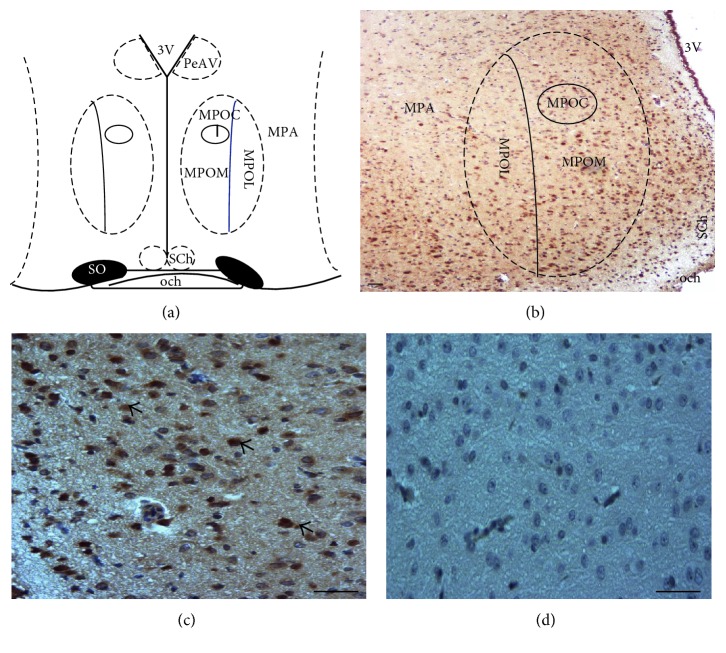
A representative diagram of the POA-AHA region shows the site of microinjections and where cell counting was performed. The diagram is based on the Paxinos and Watson's stereotaxic atlas for the rat brain (a). Representative photomicrographs showed the immunoreactive neurons (brown) for m_2_AChR of intact rats on the right side of the POA-AHA, where cells were counted within the central, lateral, and medial portions of the medial preoptic nucleus, magnification 10x (b) and 40x (c); arrowheads show the immunostained (cytoplasmic) neurons in the POA-AHA region. Negative control, magnification 40x (d). Scale bar = 50 *μ*m. See Section 2 for details. MPA, medial preoptic area; MPOM, medial part of medial preoptic nucleus; MPOL, lateral part of medial preoptic nucleus; MPOC, central part of medial preoptic nucleus; PeAV, paraventricular nucleus; 3V, third ventricle; SCh, suprachiasmatic nucleus; SO, supraoptic nucleus; och, optic chiasm.

Intact rats in each phase of the oestrous cycle were anesthetized with sodium pentobarbital and sacrificed at 09:00, 13:00, or 17:00 h (3 rats per time point). These time points were selected because previously our group has reported several findings about the participation of Ach in the ovulation and its role in the secretion of the sex steroid hormones in those critical hours [7–13]. The brains were removed and placed in 4% paraformaldehyde solution for 24 h, dehydrated with graded alcohol, and embedded in paraffin. Immunoreactive neurons for m_1_AChR (m_1_AChR-ir) or m_2_AChR (m2AChR-ir) were detected using a conventional avidin-biotin immunoperoxidase protocol. Brain sections (10 *μ*m thick) were deparaffinized, hydrated, and pretreated with 1% H_2_O_2_ for 30 min to quench endogenous peroxidase activity; rinsed in 0.1 M phosphate buffer; and then incubated in 1% NaBH_4_ (Sigma Chemical Co.) to reduce free aldehydes. For antigen retrieval, 10 mM sodium citrate buffer was used. The tissue sections were incubated at 4°C for 48 h with either m_1_AChR or m_2_AChR antibodies (sc-9106 or sc-9107, Santa Cruz Biotechnology Inc., Dallas, TX) (1 : 25 dilution). Next, the sections were incubated with secondary polyclonal antibodies (1 : 100 dilution) for 3 h at room temperature (pk-6101, VECTASTAIN Elite ABC Kit, Vector Laboratories Inc., Burlingame, CA). Immune complexes were detected with the avidin DH-biotinylated horseradish peroxidase H complex, and brown immunostaining was observed in the cytoplasmic compartment. The sections were counterstained with Mayer's haematoxylin to stain the nucleus (blue/purple) and dehydrated permanently in nonaqueous mounting media. As a negative control, the primary antibody was omitted. The number of m_1_AChR-ir or m_2_AChR-ir neurons was determined by counting positive cells (brown immunostaining) as described by Mendoza-Garcés et al. [15]. The positive cells were counted within the central, lateral, and medial portions of the medial preoptic nucleus (Figure 1) using a light microscope with a KS300 imaging system (Carl Zeiss, Germany). The region was restricted to rostral-caudal coordinates −0.6 to −0.68 mm relative to the bregma of the left or right side of the POA-AHA region [16].

### 2.3. Effects of the Blockade of m_1_AChR or m_2_AChR in the Left or Right POA-AHA on Spontaneous Ovulation

**Figure 2:**
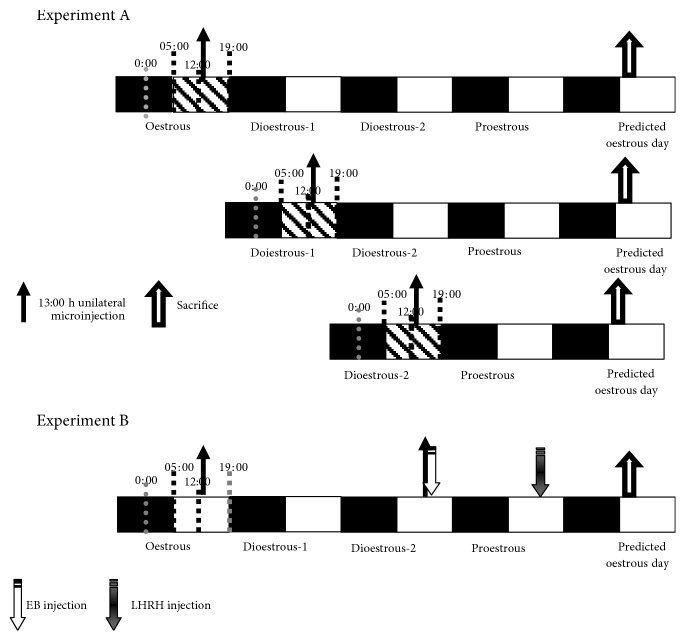
Schematic representation of the experiments A and B. Experiment A: experimental design for unilaterally microinjecting drugs (PZP, MTC, or vehicle) on the left or right side of the POA-AHA region on the day of oestrous, dioestrous-1, or dioestrous-2. Experiment B: experimental design for re-establishing the hypothalamic signal through the replacement of LH-releasing hormone (LHRH) or oestradiol benzoate (EB). All animals were sacrificed on the predicted day of oestrous.

The time line for the two experimental paradigms of this study is diagrammed in Figure 2. The animals were anesthetized with pentobarbital (25 mg/kg; Anestesal, Smith-Kline, Mexico City) and placed in a stereotaxic apparatus (David Kopf Instruments, Tujunga, CA). The skin of the skull was sectioned, and the left or right side of the skull was drilled with a 1 mm bit. Subsequently, a 29-gauge stainless steel needle was lowered into the left or right side of the POA-AHA. The POA-AHA was located using the bregma coordinates from the atlas as the reference (A-P, 0.679 to 0.628; lateral, 0.06; and vertical, 0.86) [16], following a previously described protocol [9–12]. The needle was connected to a 20 *μ*L Hamilton syringe placed on a microinjection pump (CMA/100; BAS, Stockholm, Sweden) with a Teflon tube (0.65 mm OD 9, 0.12 mm OI; Bioanalytical Systems Inc., West Lafayette, IN).

Groups of rats (*n* = 8–10 rats per group) at oestrous, dioestrous-1, or dioestrous-2 (Figure 2) were unilaterally microinjected in the left or right POA-AHA region with the following: (a) 1 *μ*L of vehicle (0.9% saline *v*/*v*); (b) 100 pg/*μ*L of pirenzepine dihydrochloride (PZP) (Sigma-Aldrich, Mexico), an m_1_AChR antagonist; and (c) 100 pg/*μ*L of methoctramine (MTC) (Sigma-Aldrich, Mexico), an m_2_AChR antagonist. All solutions were injected at a rate of 1 *μ*L/min. Vaginal smears were taken 24 h after surgery, and the animals were sacrificed at 10:00 h on the next predicted day of oestrous; then, the oviducts were dissected, the number of ova shed was counted with a stereo microscope (Olympus SZ51-LGB, Tokyo, Japan), and the ovulation rate was analysed. A different group of untreated (intact) animals was sacrificed at 10:00 h on the day of vaginal oestrous for control purposes. Since pentobarbital injection at 13:00 h on the proestrous day blocks ovulation [17, 18] and the LHRH surge [19], we did not study the effects of blockade of m_1_AChR or m_2_AChR on the proestrous day.

### 2.4. Effects of the Replacement of LHRH or EB in Rats with Blockade of m_1_AChRs or m_2_AChRs in the Left or Right POA-AHA on Spontaneous Ovulation

Other groups of animals (*n* = 5–8) treated with PZP or MTC on either side of the POA-AHA region were subcutaneously (s.c.) injected with 3.7 *μ*g/kg of synthetic LH-releasing hormone (LHRH-Gly-OH) at 14:00 h on the day of proestrous or with 10 *μ*L of oestradiol benzoate (EB) at 14:00 on dioestrous-2 (Sigma Chemical Co., St. Louis, MO). The animals were sacrificed on the morning of the next predicted day of oestrous, and the ovulation rate was analysed (Figure 2).

### 2.5. Brain Histological Procedures

To verify the accuracy of the microinjection site, 100 *μ*m sections of the POA-AHA region were obtained with a vibratome (Technical Products International Inc., St. Louis, MO, USA). The sections were mounted on slides and were immediately examined under a stereoscopic microscope. Only rats with verified microinjection into the POA-AHA were used in the study.

### 2.6. Statistical Analyses

Data for m_1_AChR-ir or m_2_AChR-ir cells were analysed by one-way analysis of variance (ANOVA) followed by Tukey's multiple comparison test. All measurements are expressed as the mean ± SEM. Data on the ovulation rate (number of ovulating animals over total number of the treatment group) were analysed using the chi-square test. Data on the number of ova shed were analysed using the Kruskal-Wallis test followed by Dunn's test. A probability value of *p* ≤ 0.05% was considered significant. All statistical analyses were performed with GraphPad InStat3 Software Inc. (San Diego, CA, USA).

## 3. Results

### 3.1. Changes in the Number of m_1_AChR-ir or m_2_AChR-ir Neurons in Each Side of the POA-AHA throughout the Oestrous Cycle

**Figure 3:**
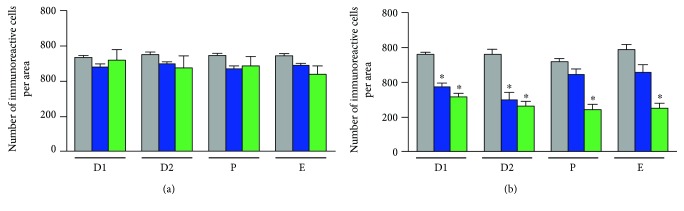
Number of m_1_AChR or m_2_AChR immunostained neurons (m_1_AChR-ir or m2AChR-ir) in the POA-AHA region throughout the oestrous cycle in the rat. A number of m_1_AChR-ir (a) or m_2_AChR-ir (b) were counted on both sides (left side plus right side) of the medial preoptic nucleus in the preoptic region at 9:00 (grey bar), 13:00 (blue bar), or 17:00 h (green bar) for each phase of the oestrous cycle. The results are expressed as the mean ± SEM. ^∗^*p* < 0.05 versus 9:00 h, for its respective phase of the oestrous cycle. D1, dioestrous-1; D2, dioestrous-2; P, proestrous; E, oestrous.

No significant changes were observed in the number of m_1_AChR or m_2_AChR positive cells between the left and right sides of the POA-AHA region at 9:00, 13:00, or 17:00 h on each day of the oestrous cycle. However, we observed that the number of m_2_AChR positive cells on both sides (left side plus right side) of the POA-AHA region was significantly higher at 09:00 h of each day of the oestrous cycle than at 13:00 or 17:00 h (Figure 3).

### 3.2. Effects of the Blockade of m_1_AChRs or m_2_AChR in the Left or Right POA-AHA on the Ovulation Rate

**Figure 4:**
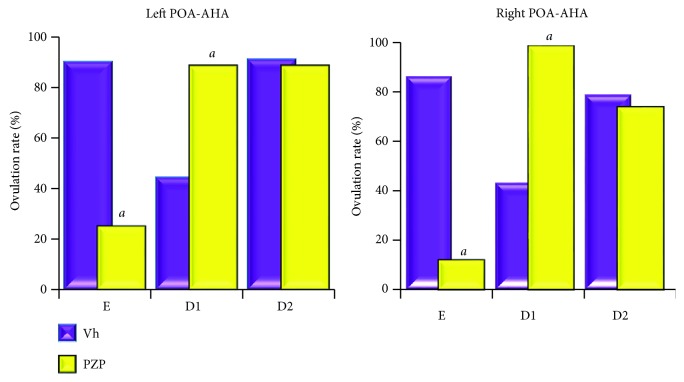
The effects of vehicle (Vh) or pirenzepine (PZP) microinjected in the left or right side of the POA-AHA region on ovulation. The ovulation rate (number of ovulating animals over total number of the treatment group) of rats microinjected with vehicle (Vh) or pirenzepine (PZP) in the left or right POA-AHA at 13:00 h of the oestrous (E), dioestrous-1 (D1), or dioestrous-2 (D2). ^*a*^*p* < 0.05 versus the respective Vh group (chi-square test).

**Figure 5:**
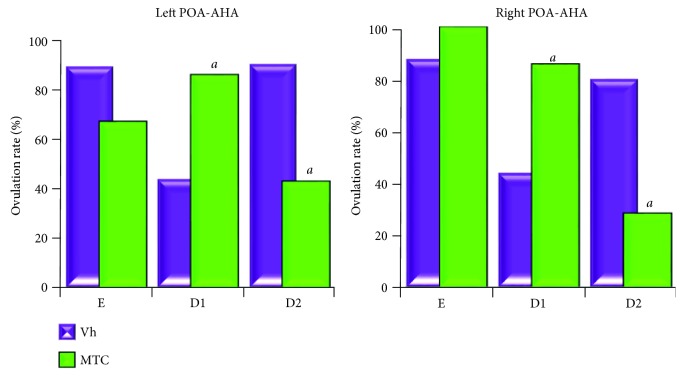
The effects of vehicle (Vh) or methoctramine (MTC) microinjected in the left or right side of the POA-AHA region on ovulation. The ovulation rate (number of ovulating animals over total number of the treatment group) of rats microinjected with vehicle (Vh) or methoctramine (MTC) in the left or right POA-AHA at 13:00 h on the day of oestrous (E), dioestrous-1 (D1), or dioestrous-2 (D2). ^*a*^*p* < 0.05 versus the respective Vh group (chi-square test).

In comparison with that in the vehicle group, the microinjection of PZP on either side of the POA-AHA on the day of oestrous (Figure 4) or MTC on dioestrous-2 (Figure 5) resulted in a lower ovulation rate. The ovulation rate in animals microinjected with the vehicle on dioestrous-1 was lower than that in the intact group (left POA-AHA: 4/12 or right POA-AHA: 4/10 versus control: 10/10; *p* < 0.01), while PZP or MTC microinjection restored ovulation on dioestrous-2 (Figures 4 and 5).

The number of ova shed from both ovaries of rats microinjected with PZP or MTC on either side of the POA-AHA on dioestrous-2 was lower than that of those microinjected with the vehicle (PZP: 6.2 ± 0.9 or MTC: 6.2 ± .4 versus vehicle: 11.5 ± 0.7; *p* < 0.001). Interestingly, when the number of ova shed was counted per ovary, we observed that animals microinjected in the right POA-AHA with PZP on dioestrous-2 released lower numbers of oocytes than those treated with vehicle (left ovary: 3.8 ± 0.9 versus 6.9 ± 0.3, *p* < 0.0025; right ovary: 0 versus 5.9 ± 0.4). This effect was not seen in the animals that received microinjections into the left POA-AHA region.

### 3.3. Effects of Hormonal Replacement with LHRH or EB on Nonovulating Rats with Blockade of m_1_AChRs or m_2_AChR in the Left or Right POA-AHA

The LHRH or EB replacement therapy for nonovulating rats unilaterally microinjected with PZP or MTC on either side of the POA-AHA region restored ovulation in all treated rats.

## 4. Discussion

The results of the present study suggest that in the POA-AHA region, ACh regulates spontaneous ovulation through the m_1_AChR and m_2_AChR and this regulation depends on the oestrous cycle phase. In oestrous, ovulation is regulated by m_1_AChR, while dioestrous-2 is regulated by m_2_AChR. The fact that ovulation is blocked by changes to the binding of ACh to m_1_AChR or m_2_AChR and that the activation of these receptors is dependent on the day of the oestrous cycle suggests that the activation of each receptor results in different intracellular signals necessary for the regulation of ovulation. The m_2_AChRs are selectively coupled to the Gi/Go family of G proteins [20]. Since m_2_AChRs mediate the inhibition of voltage-sensitive Ca^2+^ channels [21] that are known to be intimately involved in the regulation of neurotransmitter release, m_2_AChRs are considered the major inhibitory muscarinic autoreceptors in the mouse hippocampus and cerebral cortex [22]. In brain slices from adult male mice, blocking endoplasmic reticulum calcium reuptake to elevate intracellular calcium evokes GnRH release in both the median eminence and preoptic area (POA) [23]. Therefore, the blockade of m_2_AChR on the POA-AHA could increase the release of ACh and could elicit GABAergic transmission, as occurred in primary cultures of the lateral hypothalamus via nicotinic pathways [24], which in turn would inhibit GnRH secretion [25]. In vitro studies have shown that an increase in intracellular calcium induced by Ach could be modulated by oestradiol in LHRH neurons through specific receptor sites at the plasma membrane [26]; these results could help to partially explain how oestradiol exerts its rapid, negative feedback actions on GnRH and LH secretion in female reproduction. Twenty years ago, Sokolovsky et al. [27] suggested that the muscarinic receptors play a role in the positive or negative (or both) regulation of the oestrogens on sex hormone secretion. Previous results by our group had shown that the unilateral microinjection of vehicle on the right side of the POA-AHA at 09:00 h on dioestrous-1 reduces the ovulation rate [13]. A similar effect was observed in the present study when either side of the POA-AHA was microinjected with the vehicle. This could be attributable to the different reactivities of neurons or glia to the neuroendocrine signals generated as a consequence of the swelling process induced by the vehicle treatment [28]. Cytokines released during vehicle-induced swelling or during the inflammatory process would directly or indirectly affect GnRH secretion [29, 30]. Another possible explanation is that a local increase in ACh was induced by the vehicle microinjection, suggesting that on dioestrous-1, the ACh attached to the m_1_AChR or m_2_AChR inhibits the process of ovulation, since the blockade of m1 or m2 restores ovulation.

The blockade of the m_2_AChR in either side of the POA-AHA on dioestrous-2 decreased spontaneous ovulation, so we postulate that at 13:00 h on dioestrous-2, the activation of these receptors by ACh plays a stimulatory role in the mechanisms regulating ovulation. The low number of released ova from rats with m_1_AChR blockade on dioestrous-2 suggests that the stimulation of these receptors is also necessary for normal ovulation. We have previously shown that the unilateral blockade of mAChR in the POA-AHA on dioestrous-1 or dioestrous-2 by implants of atropine slows follicular growth [13]. Marchetti et al. [31] proposed the existence of a direct neural connection between the brain and the ovaries, and our results support such an idea and suggest that this neural connection could be through the activation of muscarinic receptors located in the POA-AHA region.

In contrast, on the day of oestrous, activation of m_1_AChR is required for ovulation. Activation of m_1_AChR leads to the Gq protein-mediated activation of phospholipase C, which causes the formation of inositol 1,4,5-trisphosphate (IP_3_). Formed IP_3_ releases Ca^2+^ from Ca^2+^ stores in the endoplasmic reticulum [20] and then causes the release of GnRH [23]. Therefore, blocking these receptors inhibits the secretion of GnRH [25] and LH, thus inhibiting ovulation. Previous reports have shown that activation of m_1_AChR in GT1-7 cells (obtained from a hypothalamic tumour in a transgenic mouse) by ACh leads to the stimulation of phosphoinositide hydrolysis, which is followed by increased LHRH secretion [32]. Morales et al. [33] suggested a rapid effect of oestradiol on ACh-induced calcium signals in GT1-7 cells through cyclic GMP cascade. Interestingly, the maximal response of GT1-7 cells to ACh on calcium mobilization occurs at 5 seconds, and ACh-induced calcium transients were blocked completely by atropine [23], suggesting that the decrease in cytosolic Ca^2+^ accumulation could be resulted of the blockade of m_1_AChR or the blockade of ACh release through an inhibitory muscarinic autoreceptors. In the case of the blockade of m_2_AChR, it could result in the absence of secretion of GnRH and LH and thus ovulation. Interestingly, whatever signaling mechanism is triggered in the GnRH network, ACh bound to muscarinic receptors plays a stimulatory role in ovulation.

The specific blockade of m_1_AChR or m_2_AChR on each portion (right or left side) of the POA-AHA does not regulate ovulation in an asymmetric way, as occurred when all the mAChRs (m_1_–m_5_) were blocked at the same time by atropine implants [9]. Since, at 13:00 h of each day of the oestrous cycle, the number of m_1_AChR-ir and m_2_AChR-ir cells is similar on each side of the POA-AHA, we think that the binding parameters (Bmax and Kd) of the m_1_AChR and m_2_AChR to ACh could be different on each side of the POA-AHA. We have previously shown that in membranes of the left side of the POA-AHA obtained from rats at 13:00 h on dioestrous-2, the number of binding sites (Bmax) for [^3^H]-N-methyl-scopolamine is 50% lower than that in those of the right portion, but the dissociation constant (Kd) is 118% lower, and low levels of ACh were observed [34]. In the current study, we did not observe a relationship between the number of m_1_AChR-ir and m_2_AChR-ir cells and the ability of the blockade of either receptor to modify spontaneous ovulation. The differences observed in the effects of m_1_AChR and m_2_AChR blockade on ovulation performed at dioestrous-2 or oestrous could result from differences in the affinity of each mAChR present in each side of the POA-AHA.

From the effects of the unilateral microinjections of PZP and MTC on ovulation, we suggest that the number and affinity of m_2_AChR are higher on dioestrous-2 than on the day of oestrous, while m_1_AChR predominates on the day of oestrous, even when the number of cells positive for mAChR is unchanged at 13:00 h on dioestrous-2 and on the day of oestrous.

The acute microinjection of m_1_AChR or m_2_AChR selective antagonists on the regulation of spontaneous ovulation showed different effects than that previously reported in a study of chronic treatment with an atropine implant in the POA-AHA region [9]. The differing results could be explained by the following differences between the methodologies used: (1) atropine is a nonselective blocker of muscarinic receptors, and therefore, the results of the atropine implant suggest that the participation of each side of the POA-AHA in the regulation of GnRH secretion is asymmetric and varies throughout the oestrous cycle; (2) PZP and MTC antagonize only one receptor type, suggesting that the effects of ACh binding to each receptor have a different participation in the regulation of GnRH secretion; (3) the concentrations of the drugs used in the experiments are very different (100 pg in the current experiment and 25 ± 3 *μ*g on implanting atropine research); and (4) the microinjection of the antagonist in solution produces a short-lived effect because the cerebrospinal fluid flow washed away the drug that was not binding to the receptor, while the atropine crystal implants stayed longer, producing a longer-lasting effect than the microinjections.

The number of ova shed reduced from each ovary in rats that were microinjected with 100 pg/*μ*L of PZP or MTC on dioestrous-2; these results suggest that ACh regulates the growth of ovarian follicles through m_1_AChR or m_2_AChR. Interestingly, we have previously shown that at this stage of the cycle, implants of atropine in the POA-AHA region produce low follicular growth and atresia in the left ovary, without any apparent effects on the right ovary [13].

LHRH- or EB-induced ovulation observed in rats treated with PZP or MTC in the POA-AHA region supports the idea that inhibition of the LH or GnRH surge results from the inhibition of GnRH or oestradiol secretion, respectively, and/or from the stimulating feedback effects of oestradiol on LH release.

## 5. Conclusion

Based on the present results, we suggest that ovulation requires the stimulation of the m_1_AChR on either side of the POA-AHA region on the day of oestrous and the stimulation of m_2_AChR on dioestrous-2, while on dioestrous-1, the activation of both receptors inhibits the mechanisms that regulate the spontaneous ovulation process. Therefore, m_1_AChR and m_2_AChR activation is also required on dioestrous-2 for a complete restoration of ovulation.

## Additional Points

All data sets on which the conclusions of the paper rely are available to readers.
